# Hypotensive Dumping Syndrome in Hypertrophic Obstructive Cardiomyopathy

**DOI:** 10.31662/jmaj.2024-0194

**Published:** 2024-11-01

**Authors:** Takahiko Kinjo, Shogo Hamaura, Hirofumi Tomita

**Affiliations:** 1 Department of Cardiology and Nephrology, Hirosaki University Graduate School of Medicine, Hirosaki, Japan

**Keywords:** hypertrophic obstructive cardiomyopathy, dumping syndrome, distributive shock, nasogastric tube malposition

A 79-year-old woman with hypertrophic obstructive cardiomyopathy (HOCM) (Figure 1) was admitted for pneumonia (Figure 2) and require intubation and mechanical ventilation. Severe hypotension with restlessness was reproducibly induced 30 min after feeding using a nasogastric tube. We diagnosed the patients with early dumping syndrome because gastrointestinal radiography revealed that the nasogastric tube tip was positioned at the gastric pylorus (Figure 3), whereas the duodenum was immediately contrasted. Post-feeding hypotension no longer occurred after the tube tip was pulled back into the gastric body. Dumping syndrome without previous gastric surgery was also reported when a nasogastric tube ^(1)^ or gastrostomy tube ^(2)^ was positioned in the duodenum.

**Figure 1.**
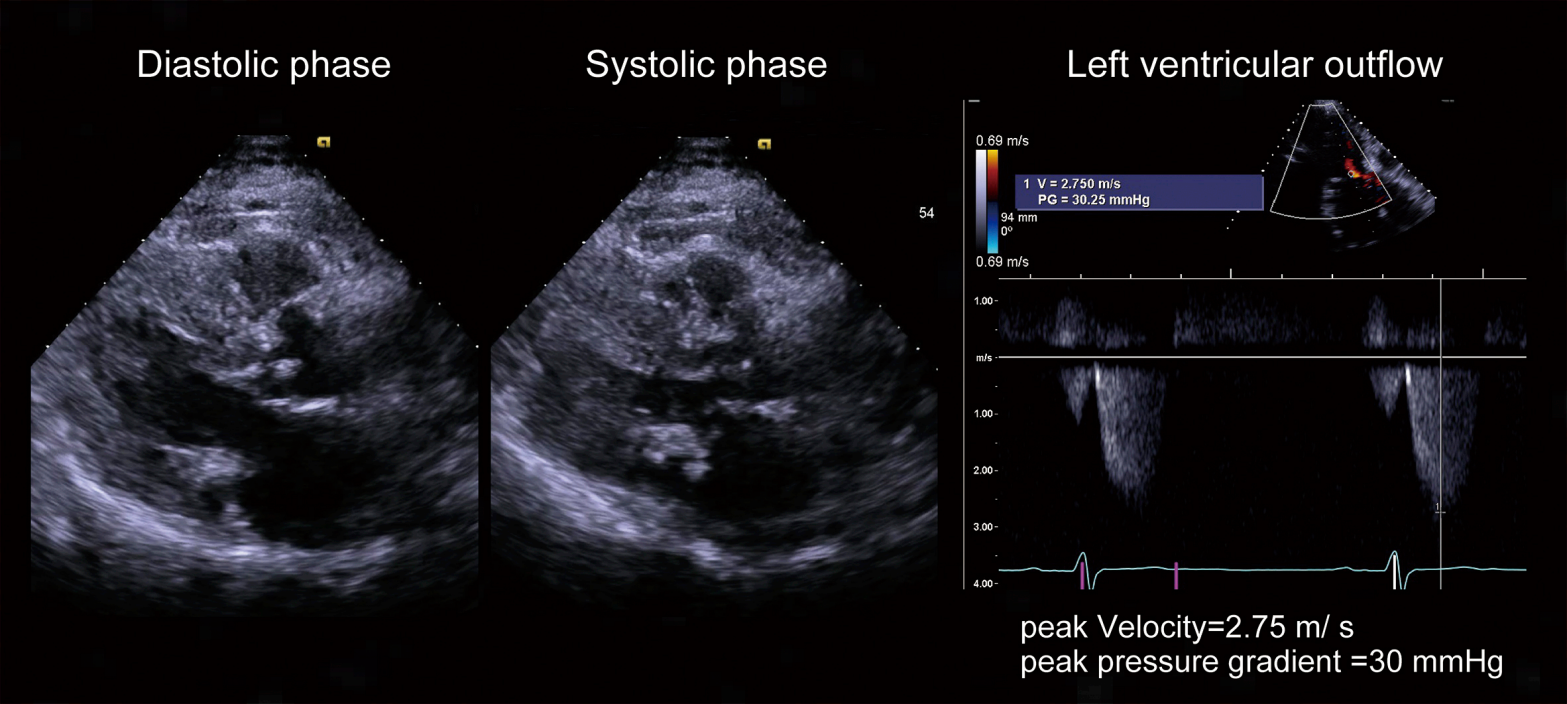
Echocardiography of the patient. The patient exhibited asymmetric septal hypertrophy; the interventricular septum measuring 16 mm and the left ventricular posterior wall measuring 9.1 mm (left and middle panel). Additionally, increased blood flow through the left ventricular outflow tract confirmed the diagnosis of obstructive hypertrophic cardiomyopathy (right panel).

**Figure 2.**
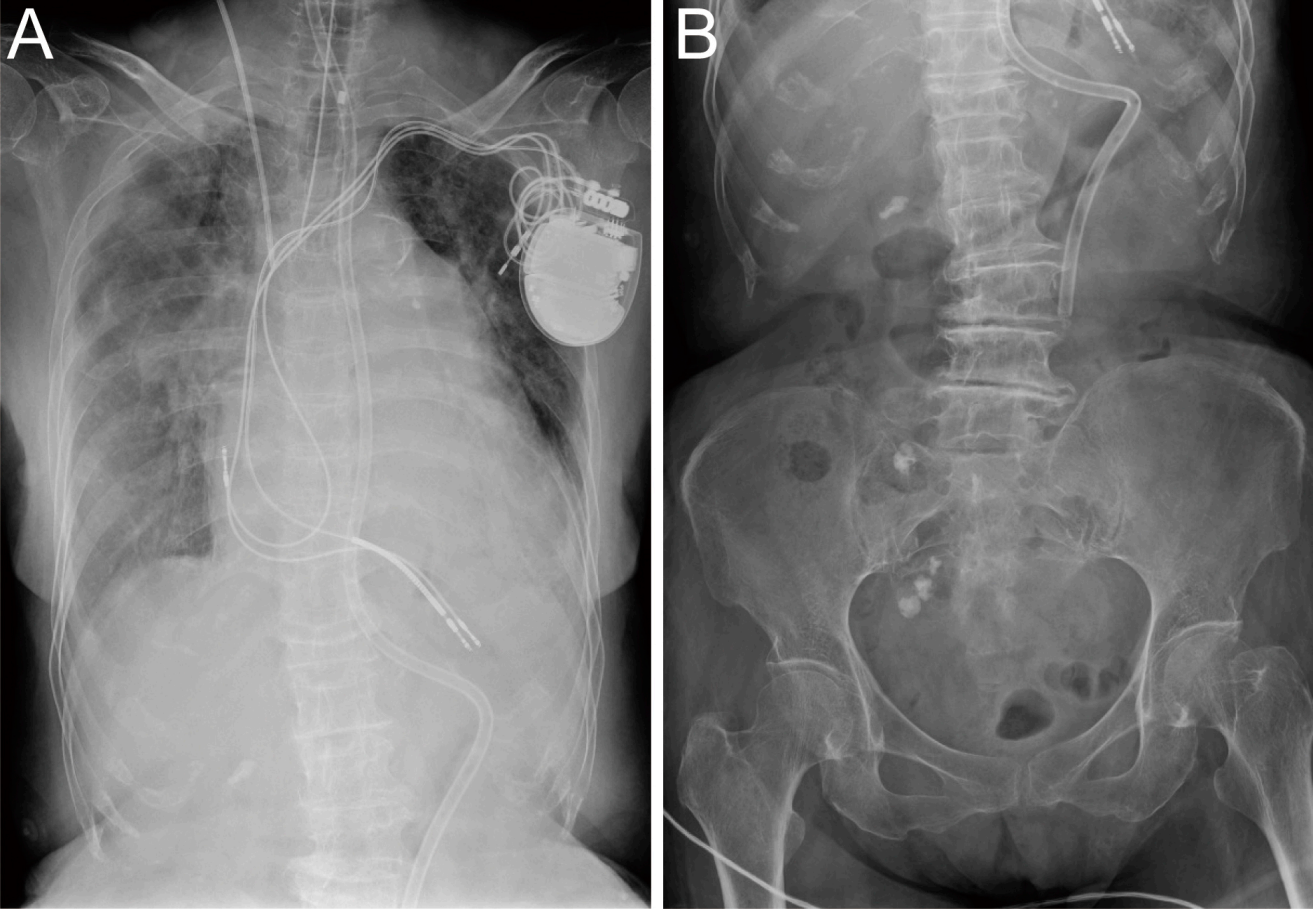
Radiography at the admission. A: Chest radiography revealed lung consolidation. Note that the patient is endotracheally intubated and a nasogastric tube is inserted. B: Abdominal radiography. The nasogastric tube tip was beyond the diaphragm, and the tube was fixed at the nasal cavity at 65 cm. Note: the height of the patient is 153 cm.

**Figure 3.**
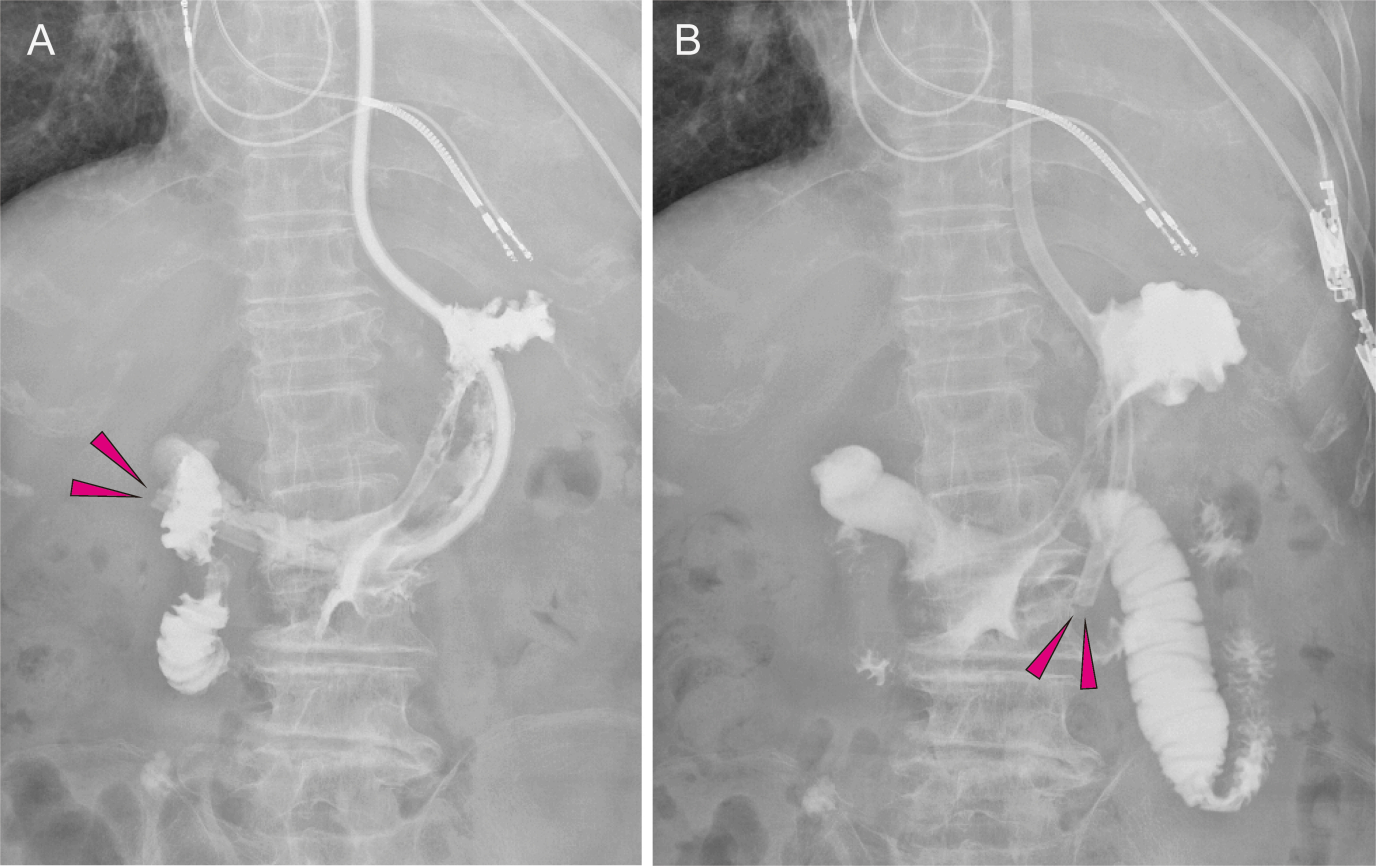
A: Gastrointestinal radiography using a nasogastric tube. The nasogastric tube tip (arrows) is positioned at the gastric pylorus, whereas the duodenum is immediately in contrast. The tube tip position progressed from the initial position, presumably via peristalsis. B: The nasogastric tube tip (arrows) was pulled back into the gastric body. The tube was adjusted and fixed in the nasal cavity at 55 cm.

Reduction of preload and vasodilation exacerbates left ventricular outflow obstruction in patients with HOCM ^(3)^. Early dumping syndrome, which is characterized by rapid flushing of the hyperosmolar feed into the intestine and subsequent fluid distribution and vasodilation, may cause severe hypotension in patients with HOCM.

## Article Information

### Conflicts of Interest

Dr. Hirofumi Tomita is a concurrent professor of the Department of Advanced Management of Cardiac Arrhythmias and received a research grant from Abbott Medical Japan LLC. Other authors have no relevant disclosures.

### Author Contributions

TK conceived the study and wrote the manuscript. All authors contributed to patient care and to editing the manuscript.

### Approval by Institutional Review Board (IRB)

An IRB approval is not required because this is a case report.

### Patient Consent Statement

The patient gave informed consent to publish this case report.

### Data Availability Statement

Data sharing does not apply to this article because no datasets were generated or analyzed during this report.
